# Editorial for the Special Issue on Recent Advances in Lithography

**DOI:** 10.3390/mi17090996

**Published:** 2026-08-24

**Authors:** Sikun Li, Yayi Wei, Hanzhi Zhang, Jibin He, Xianhao Peng

**Affiliations:** 1 Shanghai Institute of Optics and Fine Mechanics, Chinese Academy of Sciences, Shanghai 201800, China; jibinhe.st@zju.edu.cn (J.H.); pengxianhao@siom.ac.cn (X.P.); 2 University of Chinese Academy of Sciences, Beijing 100049, China; 3 Institute of Microelectronics, Chinese Academy of Sciences, Beijing 100029, China; 4 School of Physics, Changchun University of Science and Technology, Changchun 130022, China; zhanghanzhi@mails.cust.edu.cn; 5 Zhongshan Institute of Changchun University of Science and Technology, Zhongshan 528400, China; 6 College of Integrated Circuits, Zhejiang University, Hangzhou 311200, China

## 1. Introduction

Integrated circuits (ICs) constitute the fundamental hardware platform for modern information technology systems, enabling critical applications such as artificial intelligence, smartphones, computing systems, robotics, aerospace technologies, and the Internet of Things [1,2,3,4,5,6]. Advances in ICs are closely tied to continued progress in semiconductor manufacturing, which enables increasing integration density and device performance. For decades, Moore’s law—which predicts that transistor density doubles approximately every 18 to 24 months—has driven the persistent scaling of device features to smaller dimensions [7], thereby imposing increasingly stringent requirements on patterning fidelity and process control. Within this framework, lithography serves as the core technology for transferring micro- and nanoscale patterns, playing a decisive role in determining critical dimensions, device performance, and manufacturing yield, while also serving as a key enabler for fabrication across micrometer to nanometer scales [8,9].

Despite continuous technological advances, several critical challenges remain in advanced process nodes. In overlay metrology, a pronounced trade-off exists among accuracy, throughput, and robustness. Conventional imaging-based approaches struggle to simultaneously achieve optimal focus, alignment, and signal quality, while chemical mechanical planarization (CMP)-induced deformation and etch non-uniformity introduce systematic errors. In computational lithography and mask optimization, a conflict persists between imaging fidelity and manufacturability: inverse lithography technology (ILT) offers high accuracy but often produces highly complex mask patterns, whereas rule-based methods are more fabrication-friendly but offer less optimization flexibility. Optical aberrations and mask three-dimensional effects further complicate the optimization process. At the levels of process integration and materials, the nonlinear coupling of multiple exposure and etching steps degrades model predictability; source fluctuations and thermal effects amplify imaging errors; and emerging materials and self-assembly techniques face challenges such as complex mechanisms and difficulties in defect control.

Consequently, advanced lithography technology is evolving into a highly interdisciplinary field integrating optical engineering, computational science, artificial intelligence, and semiconductor processes. This special issue, focusing on key scientific issues and engineering challenges in advanced lithography, gathers recent research achievements spanning overlay metrology, computational lithography, process control, lithographic materials and application of AI in these technical aspects. Based on research content and methodological characteristics, these contributions are categorized into five themes: Lithography Tool and Technology [contribution 1–5] focuses on system-level stability and control of lithography tools, covering reviews and optimizations of key technologies ranging from thermal management and source stability to projection imaging defect mechanisms, thereby providing theoretical and engineering support for enhancing ultimate equipment precision; Computational Lithography [contribution 6–9] addresses the balance between accuracy and efficiency, explores the evolution of inverse lithography technology, and proposes advanced optimization algorithms that balance mask manufacturability with imaging fidelity, effectively extending the process window for advanced nodes; Metrology [contribution 10–12] tackles challenges in overlay metrology for advanced semiconductor processes through integrated focus control and image acquisition, process-aware alignment diagnostics, and error correction based on higher-order diffraction signals, addressing the coupled demands of measurement speed, accuracy, and robustness to process-induced distortions; Materials [contribution 13,14] brings together a mechanistic study of ligand-dependent resist chemistry and a systematic review of the materials, interfaces, and process conditions shaping directed self-assembly (DSA) integration and scaling; AI Applications in Lithography [contribution 15–20] demonstrates the deep integration of artificial intelligence throughout the lithography workflow, utilizing data-driven and deep learning methods to effectively resolve computational bottlenecks and accuracy challenges in metrology, modeling, optimization, and inspection. Together, these contributions form an integrated technological chain spanning metrology, optimization, and manufacturing.

Overall, these studies advance both the mechanistic understanding of complex lithographic processes and the capability for high-precision manufacturing. Looking ahead, artificial intelligence is expected to improve the efficiency of metrology and alignment; the fusion of data-driven and physics-based models will further optimize mask design; and the co-optimization of materials, processes, and equipment will enable end-to-end process control, continuously driving semiconductor manufacturing toward higher integration, lower power consumption, and enhanced performance.

## 2. Lithography Tool and Technology

This section focuses on the imaging characteristics and technical principles of lithography tools, as well as subsystems such as thermal control, focus-leveling, and light sources. Related research includes: Cao et al. systematically reviewed the thermal control systems of deep ultraviolet (DUV) and extreme ultraviolet (EUV) projection lithography tools, summarized thermal error suppression strategies from the dimensions of hardware structure and control algorithms, and identified cross-module coordination and hybrid modeling as promising directions for future development [contribution 1]. Li et al. addressed the issue of measurement accuracy in focus-leveling sensors caused by process pattern interference by introducing the Criminisi algorithm to recover the image, successfully controlling the average repair error at the sub-nanometer level [contribution 2]. He et al. constructed a High-NA EUV lithography simulation model, revealed the relationship between phase defect signals and different defect positions and sizes, and illustrated the role of high NA in improving defect tolerance [contribution 3]. Zhu et al. established an imaging model for immersion DUV light sources, quantified the dominant contribution of laser parameter fluctuation to imaging errors, and provided a theoretical basis for light source stability control at advanced nodes [contribution 4]. Li et al. provided a comprehensive review of DMD-based optical lithography systems, elucidating their point-array essence, evaluating resolution, overlay accuracy, and throughput, and summarizing achievements from IC manufacturing to micro-scale fabrication [contribution 5]. Overall, the contributions in this section provide a comprehensive examination of lithography tools and technologies from the perspectives of thermal control, focus-leveling, high-NA EUV imaging, DUV light-source stability, and DMD-based optical lithography. These works not only deepen the understanding of lithographic imaging characteristics, error sources, and defect-response mechanisms through rigorous physical modeling, but also address key engineering bottlenecks in measurement accuracy, thermal drift suppression, source stability, and system performance through the coordinated use of algorithms and hardware, thereby providing important technical support for improving the overall capability of lithography equipment.

## 3. Computational Lithography

This section focuses on computational lithography technology and its optimization strategies, covering recent developments and applications in advanced semiconductor manufacturing, as well as algorithmic advances in computational efficiency, imaging performance, and mask manufacturability. Related research includes: Meng et al. comprehensively reviewed the application status and development history of ILT in advanced chip manufacturing, systematically summarizing mainstream algorithms such as level-set, pixelated ILT, and machine learning-assisted ILT, analyzed key barriers to industrial deployment, including high computational complexity and difficulties in mask fabrication, and pointing out future directions such as AI-driven design and GPU acceleration [contribution 6]. Liu et al. addressing the contradiction between computational efficiency and accuracy in curvilinear sub-resolution assist feature (SRAF) generation, proposing a U-Net neural network-based learned optimizer that, through offline training and online inference strategies, achieved higher final optimization accuracy while significantly reducing computational cost [contribution 7]. Wang et al. addressed aberration-induced imaging degradation in EUV lithography by establishing a Source-Mask Optimization (SMO) framework that effectively compensated for optical aberrations through the joint optimization of freeform illumination sources and mask patterns, improving critical-dimension uniformity (CDU) and exposure latitude (EL) [contribution 8]. Zhou et al. addressed the trade-off between imaging fidelity and mask manufacturability in conventional ILT by proposing a dual-stage optimization framework consisting of fidelity-oriented hierarchical optimization followed by manufacturability-aware mask synthesis, achieving improved imaging quality and mask manufacturability in simulation [contribution 9]. Overall, the contributions in this section provide a comprehensive examination of computational lithography from the perspectives of ILT development trends, SRAF generation efficiency, aberration compensation strategies, and manufacturability optimization frameworks. These studies not only provide a systematic overview of the technological evolution and current challenges of computational lithography, but also demonstrate practical approaches for balancing computational efficiency, imaging fidelity, and mask manufacturability through algorithmic innovations, such as learned optimizers and dual-stage optimization frameworks, thereby offering valuable guidance for addressing engineering challenges in advanced lithographic processes.

## 4. Metrology

This section brings together three complementary routes to more reliable overlay metrology: integrated focus and acquisition, process-aware alignment diagnostics, and higher-order diffraction correction. Their combined significance lies in treating measurement performance as a system-level outcome of coordinated optics, marks, models, and process conditions. Lan et al. advanced hardware–software integration by coupling a synchronized dual-wavelength, dual-camera architecture with a multi-task model for mark position and focal plane. The 405 nm and 940 nm channels were used to image current-layer alignment marks and previous-layer marks buried beneath an approximately 300-nm-thick SiN layer, respectively. For 200 marks on a 7 nm DRAM wafer, alignment and focusing time fell from 50 ± 3 ms to 12 ± 1 ms. Focal-plane repeatability improved from 23 ± 2 nm to 11 ± 1 nm, alongside higher contrast and signal-to-noise ratio [contribution 10]. Jiang et al. established complementary roles for Wafer Quality (WQ), which characterizes alignment-signal quality, and Alignment Position Deviation (APD), which captures position bias, in multilayer SiO_2_/SiN three-dimensional structures. Using segmented marks, multiwavelength and multipolarization sensing, and rigorous coupled-wave analysis, the study linked the directional and radial behavior of wafer-edge marks to etch profiles and sidewall asymmetry. The framework supports the use of WQ in mark and signal development, APD in overlay optimization, and both in high-volume monitoring [contribution 11]. Liu et al. combined first- and third-order signals at a single wavelength and exploited differences in the corresponding light-intensity-difference curves to correct nonlinear overlay errors. A subdivided grating increased the modeled third-order diffraction efficiency by more than sixfold, while scalar analysis indicated reduced sensitivity of higher-order information to process-induced asymmetric deformation of the bottom mark. Rigorous coupled-wave analysis and optical-path simulations showed that, for a prescribed overlay offset of 10 nm, the maximum error fell from nearly 0.5 nm to 0.02 nm. Across the examined asymmetric cases, it remained below approximately 0.09 nm [contribution 12]. Collectively, these contributions move overlay metrology beyond isolated accuracy optimization toward coordinated control of acquisition, process bias, and diffraction-related errors. Future priorities include EUV-compatible and in situ focus control, fabricated-target validation of higher-order strategies, cross-platform benchmarking, traceable uncertainty analysis, adaptive target design, and closed-loop process feedback.

## 5. Materials

This section connects two scales of lithographic materials research: ligand-dependent resist chemistry and the process integration of directed self-assembly. Together, these contributions show why materials should be evaluated across the full patterning sequence rather than by isolated sensitivity or resolution values. Liu et al. used a controlled comparison of two structurally analogous organotin carboxylates to examine relationships among ligand functionality, proposed exposure pathways, and resist performance. Bn_2_Sn(MAA)_2_ contains a reactive C=C bond, whereas Bn_2_Sn(i-BA)_2_ does not. Under 266 nm exposure, the i-BA compound required an exposure dose of 160 mJ·cm^−2^ compared with 262 mJ·cm^−2^ for the MAA compound, and both exhibited a contrast of approximately 3. Spectroscopic analyses supported ligand loss, increased Sn–O bond formation, and tin–oxygen condensation; the authors also proposed a competing olefin-crosslinking pathway in the MAA system. Both resists resolved 90 nm half-pitch patterns with line-edge roughness below 5 nm, and the MAA resist enabled pattern transfer to a depth of approximately 100 nm in silicon [contribution 13]. Cheng et al. provided an integrated synthesis of DSA research spanning materials, guiding interfaces, annealing, pattern transfer, modeling, defect control, and applications. The review organizes the field around graphoepitaxy and chemoepitaxy and highlights the role of high-χ block copolymers in enabling smaller domains. It also identifies molecular-weight distribution, interfacial energetics, ordering kinetics, and etch selectivity as coupled design considerations. The review further contrasts dry and wet pattern transfer and summarizes applications in pitch multiplication, roughness rectification, and dimensional uniformity [contribution 14]. These contributions extend material evaluation from molecular design to the full patterning stack. Future work should enhance resist sensitivity without compromising film or transfer performance and broaden systematic studies of ligand-dependent reaction pathways. DSA priorities include defect control, metrology throughput, and predictive structure–process–performance relationships for device integration.

## 6. AI Applications in Lithography and Metrology

This section demonstrates the deep integration of artificial intelligence across the entire lithography workflow, covering key applications such as metrology, etching modeling, mask optimization, and defect detection. Xu et al. reviewed AI-driven optical metrology technologies, systematically summarizing paradigms such as forward surrogate models, inverse prediction, and physics-informed neural networks, analyzed key challenges, including distribution shifts between simulated and measured data and cross-tool matching, and pointing out the development direction of fusing data with physical models [contribution 15]. Gong et al. addressed the nonlinear coupling challenge of dual lithography-etch cycles in multiple patterning technology, proposing an AI-assisted composite etch model that embeds a neural network as a supplementary term into the traditional modeling process, reducing the RMS error of the final etch contour prediction and supporting automatic retargeting optimization [contribution 16]. Lin et al. focused on addressing the difficulty of geometric parameter prediction in SRAF generation, developed a two-stage Unet framework that predicts SRAF centroid coordinates and dimensions step-by-step, directly outputting rectangular shapes that meet manufacturing requirements, and significantly reducing pattern error and edge placement error [contribution 17]. Wang and Ren proposed a lightweight EAAUnet-ILT framework that integrates a hybrid adaptive attention module and introduces an SRAF constraint algorithm, significantly reducing mask manufacturing shot count and complexity while ensuring imaging quality through iterative optimization [contribution 18]. Mohammad et al. utilized transfer learning to fine-tune a VGG-16 model, achieving high-precision reconstruction of EUV multilayer defect profile parameters relying solely on a small-sample dataset, effectively solving the problems of scarce training data and slow convergence in deep learning [contribution 19]. Lin et al. improved the YOLOv5 model by embedding a spatial attention mechanism and designing a data augmentation strategy based on layout flipping, effectively overcoming the sample imbalance problem in hotspot detection and achieving a comprehensive performance improvement of zero missed detections and high precision [contribution 20]. Overall, this section demonstrates the broad applicability of AI technology across the entire lithography process of metrology, modeling, optimization, and inspection. Through the fusion of data-driven approaches and physical models, the related research effectively resolves key challenges in computational efficiency, model accuracy, and sample scarcity, thereby accelerating the transition toward more intelligent and data-driven lithography workflows.

In conclusion, we sincerely thank all the authors for their contributions to the success of this Special Issue, “Recent Advances in Lithography.” We also thank the reviewers for their constructive comments, which helped improve the quality of the published papers.
